# Radiologic Considerations in Heterotaxy: The Need for Detailed Anatomic Evaluation

**DOI:** 10.7759/cureus.470

**Published:** 2016-01-27

**Authors:** Rohit Loomba, Parinda H Shah, Robert h Anderson, Yingyot Arora

**Affiliations:** 1 Department of Cardiology, Children's Hospital of Wisconsin; 2 Department of Radiology, Advocate Illinois Masonic Medical Center; 3 Institute of Genetics, Newcastle University; 4 Biomedical Sciences, Denver University

**Keywords:** heterotaxy, isomerism, malrotation, univentricular, single ventricle, congenital, spleen, bronchial, neural

## Abstract

So-called “heterotaxy” is a laterality defect characterized by isomerism of the thoracic organs and random arrangement of the abdominal organs. These findings go beyond anatomic curiosity and have functional implications. It is, thus, of the utmost importance to be able to properly identify these findings.

Radiologic studies can be invaluable in determining anomalies in the central nervous, pulmonary, cardiovascular, gastrointestinal, genitourinary, and immunologic systems in patients with isomerism. Here, we review findings associated with isomerism and their importance in the setting of isomerism with the aim of ensuring that radiologists effectively describe findings in these patients and that cardiologists understand the wide variety of congenital malformations that may be present.

## Introduction and background

So-called “heterotaxy” is a laterality defect characterized by isomerism of the thoracic organs and random arrangement of the abdominal organs. From the stance of the cardiologist, it is present when an intracardiac abnormality is associated with bronchial isomerism or abnormal arrangement of the abdominal organs, particularly the spleen. Heterotaxy, however, can affect any system of organs, and can be found with normal intracardiac structure. The visceral effects go beyond being simply anatomic, with functional implications involving several organ sytems, a point best demonstrated by the fact that absence of the spleen, multiple spleens, or even a normally located and solitary spleen all can be associated with abnormal splenic function in the setting of heterotaxy [1].

Historically, heterotaxy has been segregated on the basis of splenic anatomy with patients being classified has having asplenia or polysplenia. This approach is not ideal, as a non-trivial proportion of patients with heterotaxy have solitary spleens, while correlation between absence of the spleen, or multiple spleens, with findings in the other systems is far from perfect. Additionally, it is now recognized that heterotaxy is better segregated on the basis of isomerism of the atrial appendages rather than other features such as splenic anatomy as it is the atrial appendage morphology that is the most constant feature and offers the most consistent “syndromic clustering” [2-4]. Knocking out the Pitx1 or Cited-1 genes in mice, for example, has now been shown to lead to isomerism of the right atrial appendages, while knocking out the Lefty-1 or sonic hedgehog genes has been shown to lead to isomerism of the left atrial appendages [3, 5-7]. Distinguishing the subsets on the basis of isomerism is important as those with right isomerism have been demonstrated to have higher rates of infection and mortality, specifically postoperative mortality in patients with functionally univentricular hearts [8]. To date, however, it has proved difficult to visualize the atrial appendages echocardiographically, so that atrial appendage morphology often must be inferred in the context of associated findings. As already emphasized, it is also the case that some patients with heterotaxy have usual arrangement, or mirror-imagery, of the atrial appendages, although this represents less than 1% of all reported cases [9-11].

In general, patients with right isomerism have more complex cardiovascular malformations, often necessitating functionally univentricular palliation, along with absence of the spleen, totally anomalous pulmonary venous connections even if the pulmonary veins return to the heart, and intestinal malrotation. Those with left isomerism generally have less complex cardiovascular malformations, with multiple spleens, and interruption of the inferior caval vein [12, 13]. Arrhythmias can be present in both right and left isomerism [14, 15]. Thrombocytosis may also be present in either subset in those with functionally univentricular hearts [16, 17].

Patients with heterotaxy often undergo extensive medical evaluation, including imaging, it is important, therefore, for radiologists to be aware of heterotaxy and its associated findings. A thorough understanding of the manifestations of heterotaxy makes its diagnosis more likely, and thus allows for complete evaluation of associated abnormalities. This fact is also of significance, since in the past the syndromes have been described in terms of “situs ambiguus.” Simple description of the findings in the various systems removes any potential ambiguity. We provide here a review of the findings associated with heterotaxy, suggest appropriate strategies for imaging, and provide information that is vital to determining optimal clinical management.

## Review

### Cardiovascular malformations

A variety of cardiovascular malformations is to be expected in patients known to have heterotaxy. In this review, we discuss both intracardiac lesions and abnormalities of systemic and pulmonary venous return. Evaluation of intracardiac anatomy, nonetheless, is primarily the task of cardiologists, so our discussion on cardiovascular malformations will be limited.

Those with right isomerism are more likely to have a common atrioventricular junction guarded by a common valve, double inlet left ventricle, double outlet right ventricle, an absence of the coronary sinus, and left-handed ventricular topology. The totally anomalous pulmonary venous connection will always be present when patients have bilateral appendages of right morphology, but the pulmonary veins connect to an extracardiac site in half the cases. Discordant ventriculoarterial connections are also frequent, and pulmonary stenosis or atresia is to be anticipated. Those with left isomerism are more likely to have aortic stenosis, isolated atrial and ventricular septal defects, as well as bilateral connections of the pulmonary veins. Interruption of the inferior caval vein is more common in those with isomeric left atrial appendages [7]. A significant proportion will again have an atrioventricular septal defect with a common atrioventricular junction, but more usually with biventricular and mixed atrioventricular connections.

The precise intracardiac diagnosis need not be made by the radiologist, although characteristic radiologic findings of specific cardiovascular malformations may be apparent on the chest radiograph. More importantly, however, is an evaluation of the pulmonary vasculature and the lung fields. Based on the physiology resulting from the particular cardiovascular malformation, patients will either have oligemia or hyperemia noted on their chest radiograph, which can help narrow down the diagnosis and help guide clinical decision-making, particularly in the setting of heart failure. Oligemia will often be the result of pulmonary stenosis or atresia existing in the settings of tetralogy of Fallot or a double-outlet right ventricle with subaortic interventricular communication. Hyperemia, in contrast, will often be the result of lesions, such as hypoplastic left heart syndrome or double outlet right ventricle, with subpulmonary interventricular communication [18].

The chest radiograph can also be useful in helping to raise the suspicion of a totally anomalous pulmonary venous connection, particularly of the supracardiac and cardiac varieties. When the pulmonary veins are returning to the right side of the heart, this can cause enlargement of the right-sided atrium and right ventricular hypertrophy and dilation. Increased flow of blood to the lungs in this setting also leads to pulmonary and interstitial edema, producing the characteristic “snowman in a snowstorm” appearance. Obstruction of the pulmonary venous connections may result in the very sudden opacification of the lung fields bilaterally, and should prompt immediate further investigation [18-19].

**Figure 1:**
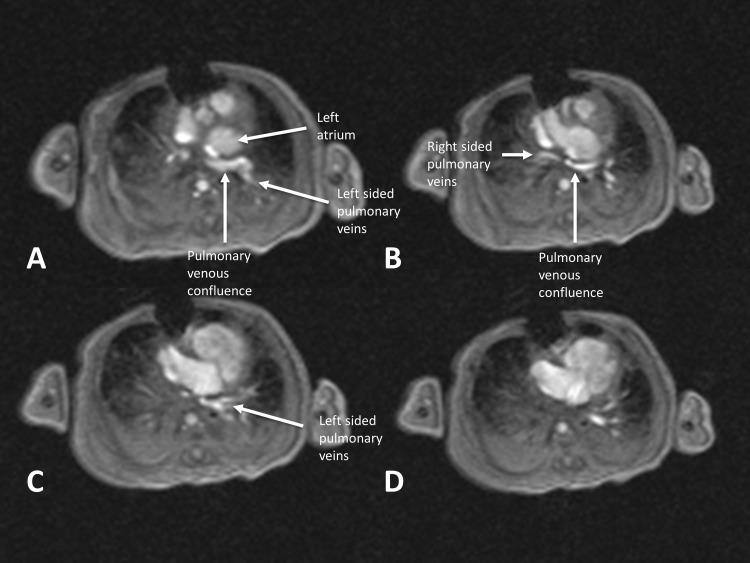
Anomalous pulmonary venous connection Magnetic resonance imaging from a single patient using a fast low angle shot (FLASH) imaging sequence in the superior to inferior direction that demonstrates totally anomalous pulmonary venous connections, with all four veins draining into a confluence posterior to the left-sided atrium.

The likely presence of anomalous pulmonary venous connections emphasizes the role of computed tomography and magnetic resonance imaging. These modalities should delineate the course of the veins and show their exact sites of cardiac or extracardiac connection. This information is important for the cardiothoracic surgeon, who needs to know if rapid intervention on the pulmonary veins is necessary. Computed tomography offers a quicker study, although at the expense of exposure to radiation while magnetic resonance imaging offers a radiation-free alternative at the expense of greater study times and the likely need for sedation in infants [19-20]. In Figure 1, we show how magnetic resonance delineated a totally anomalous pulmonary venous connection in the setting of right isomerism to a confluence that drains into the left-sided atrium.

### Bronchial and pulmonary abnormalities

As with the atrial appendages, both the bronchi and lungs are also usually isomeric in the setting of so-called heterotaxy. Van Mierop and colleagues were the first to recognize bronchial isomerism, a point later confirmed by studies done by Landing, et al. and Partridge, et al. [21-24]. Most recently, our group has confirmed their findings [25]. Bronchial isomerism is easily assessed by comparing bronchial lengths. A ratio of bronchial lengths less than 1.5 is indicative of bronchial isomerism [24]. Once bronchial isomerism is confirmed, right isomerism can be distinguished from left isomerism by measuring the bronchial angles. Angles less than 135 degrees are consistent with left isomerism while angles greater than 135 degrees indicate right isomerism. Measurements can be made on a chest radiograph or angiography in which the bronchi are visible, as well as by chest tomography or magnetic resonance imaging. Correlation between the various modalities is good, but may not always lead to the same decision. In this situation, an average of all modalities can reasonably be used. Partridge, et al. normalized bronchial length to tracheal width as a means of differentiating the isomeric subsets, although this was not confirmed in one study [24].

**Figure 2:**
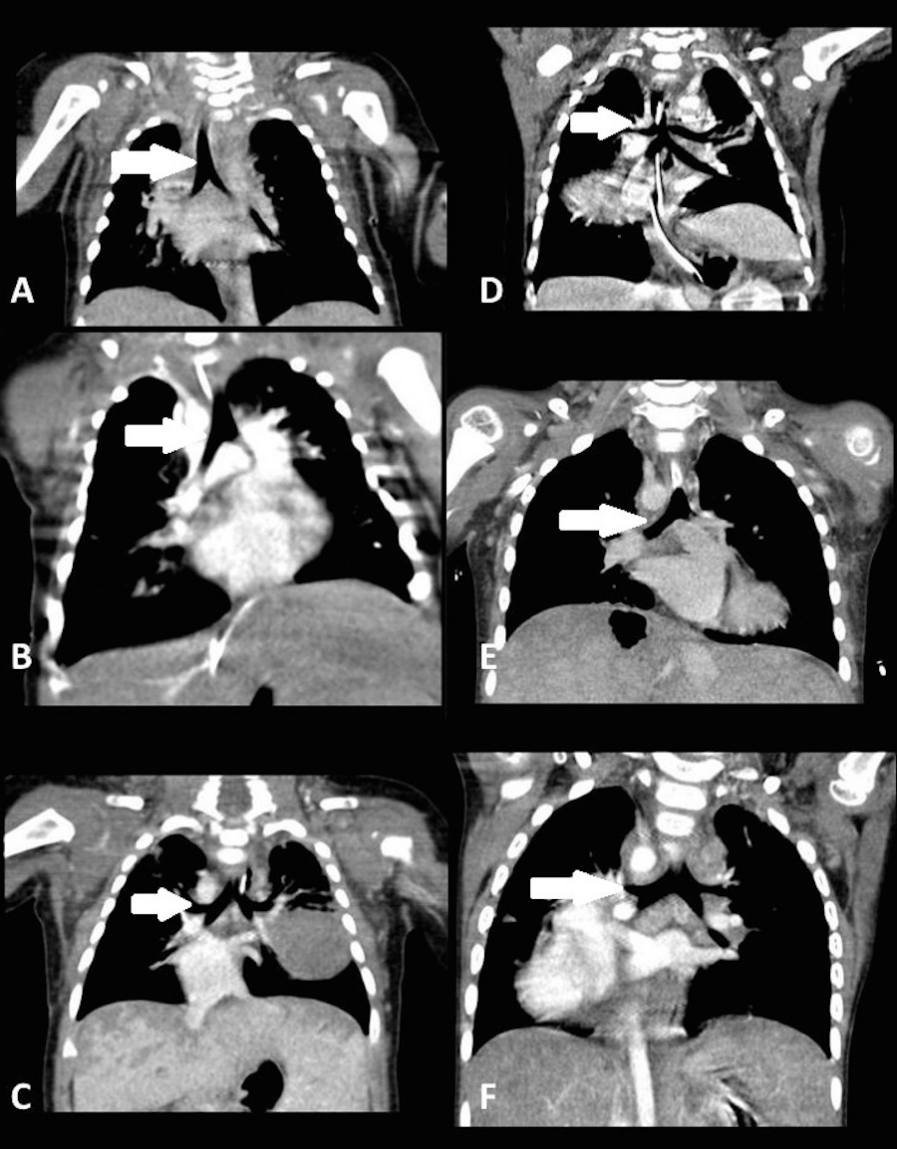
Bronchial isomerism Computed tomography of the chest in the coronal plane from six different patients demonstrating bronchial isomerism. Panels A, B, and C demonstrate bronchial angles consistent with right-sided bronchial isomerism while panels D, E, and F demonstrate bronchial angles consistent with left-sided bronchial isomerism. Arrows point to the bronchial tree.

We found the bronchial angle to be 75% sensitive and 93% specific in determining left isomerism. The negative and positive predictive value of bronchial isomerism in predicting isomerism of the left atrial appendages was 69% and 70%, respectively. In respect to determining isomerism of the right atrial appendages, right bronchial isomerism was 93% sensitive and 75% specific, with an 80% and 60% negative and positive predictive value, respectively. In Figure 2, we show examples of bronchial isomerism. In our analysis, nonetheless, we found discordances between bronchial isomerism and presumed isomerism of the atrial appendages in roughly one-fifth of patients. The clinical significance of such discordances is unclear at this time [25].

Isomerism of pulmonary lobation has also been noted, and usually is concordant with bronchial and atrial appendage isomerism. Chest radiographs, computed tomography of the chest, or magnetic resonance imaging of the chest can all be used to determine these features [26]. Direct identification of bronchial isomerism is important since it leads to further phenotypic delineation. As yet, however, it has not been established whether bronchial or pulmonary isomerism is associated with differences in respiratory outcomes.

### Immunologic abnormalities

As we have already emphasized, in the past, heterotaxy was typically segregated into the subsets of so-called asplenia and polysplenia syndromes. From the stance of cardiac lesions, it is now clear that distinction on the basis of the presumed isomerism of the atrial appendages provides a better platform for segregation. It remains crucial, nonetheless, to specifically delineate splenic anatomy, the more so since discordances with bronchial and appendage morphology are frequent. It might be assumed that splenic anatomy is important in predicting splenic function, but this proves not to be the case. Patients with heterotaxy should always be assumed to have an abnormal splenic function, since even those with normally located and solitary spleens, or multiple spleens, can have functional asplenia [27-28].

**Figure 3:**
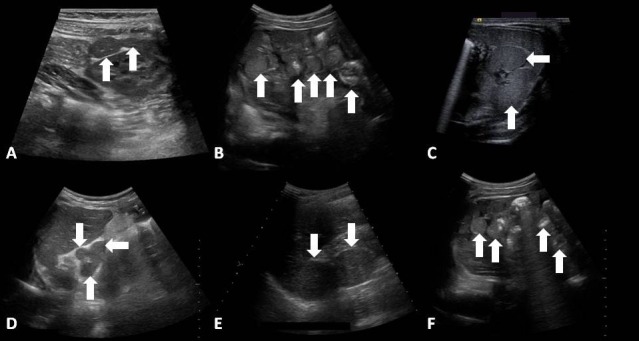
Splenic anatomy by ultrasound Abdominal ultrasound imaging from six different patients demonstrating polysplenia. Splenic tissue in the setting of heterotaxy can be found in various parts of the abdomen with a varying number of spleens.

**Figure 4:**
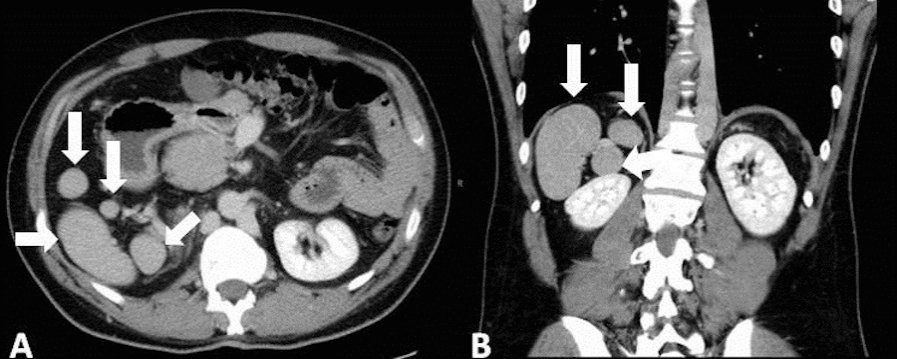
Splenic anatomy by computed tomography Computed tomography of the abdomen from a single patient demonstrating polysplenia. Panel A depicts an axial slice in which multiple well-circumscribed soft tissue masses are identified with attenuation similar to that of a normal spleen in the right posterior abdomen. Panel B depicts a coronal slice which again demonstrates multiple spleens in the right upper abdomen.

While right isomerism is more frequently associated with the absence of the spleen and left isomerism is more frequently associated with the presence of multiple spleens, this is not invariably the case [7]. Thus, documenting specific splenic anatomy is important. In infants and children, abdominal ultrasound is a reasonable modality by which to assess splenic anatomy (Figure 3) [29]. In situations where there is inadequate visualization by ultrasound, or in adults, computed tomography is a viable alternative (Figure 4) [30-31].

**Figure 5:**
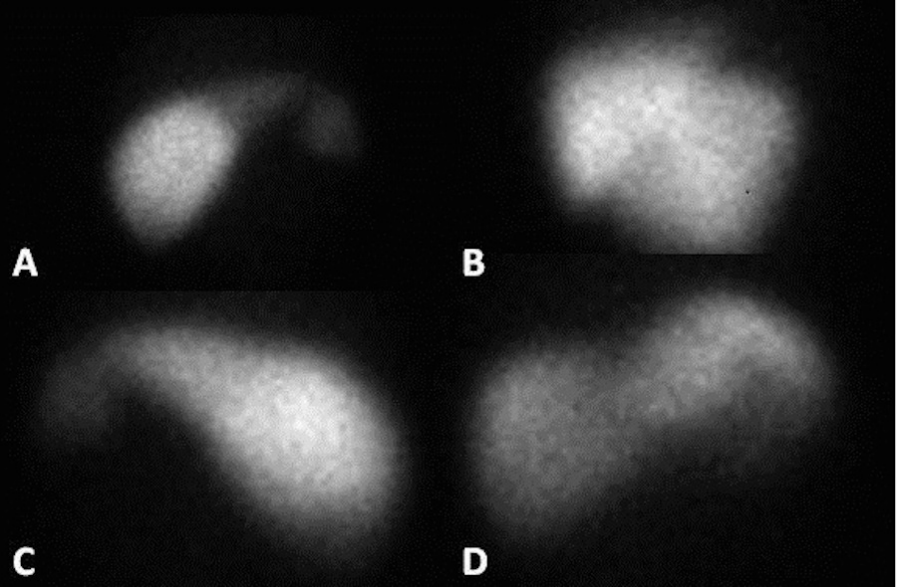
Splenic scintigraphy Splenic scintigraphy using Tc-99m sulfur colloid from a single patient in various projections demonstrating multiple spleens in the right side of the abdomen.

Yet another modality is splenic scintigraphy, which can be performed using Technetium-99m (Tc-99m)-labeled denatured erythrocytes or Tc-99m-labeled sulfur colloid (Figure 5). This technique is able to identify splenic tissue as small as 1 cm. Evaluation with Tc-99m-labeled denatured erythrocytes is preferred, as sulfur colloid can result in high liver uptake, thereby decreasing the sensitivity of the study. Splenic scintigraphy offers the advantage of being able to provide functional data in addition to anatomic data, particularly if performed with single photon emission computed tomography (SPECT)-CT [32-33]. A study comparing splenic scintigraphy with an abdominal ultrasound, nonetheless, showed that results of scintigraphy were often equivocal, and were obtained at the expense of exposure to radiation [34].

With these points in mind, therefore, an abdominal ultrasound should be the primary imaging modality used to assess splenic anatomy in infants and children. Should abdominal ultrasound not provide the necessary images, then computed tomography can be used, particularly in adults. If computed tomography offers equivocal findings, then splenic scintigraphy can be employed. For patients who undergo an abdominal ultrasound or computed tomography, the splenic function can be tested by means of identification of Howell-Jolly bodies or pitted red blood cells [35-36].

### Gastrointestinal abnormalities

In addition to abnormal lateralization of the abdominal organs, pancreatic and hepatic malformations have also been noted to be associated with heterotaxy, with some finding a frequency of malrotation approaching 70% [37]. How best to diagnose malrotation in infants is still a matter of controversy. Some would routinely evaluate the upper gastrointestinal tract while others obtain imaging only in symptomatic infants. Identification of malrotation in an otherwise asymptomatic infant then leads to further debate as to the need for a prophylactic Ladd’s procedure [38]. Such details are beyond the scope of our review.

**Figure 6:**
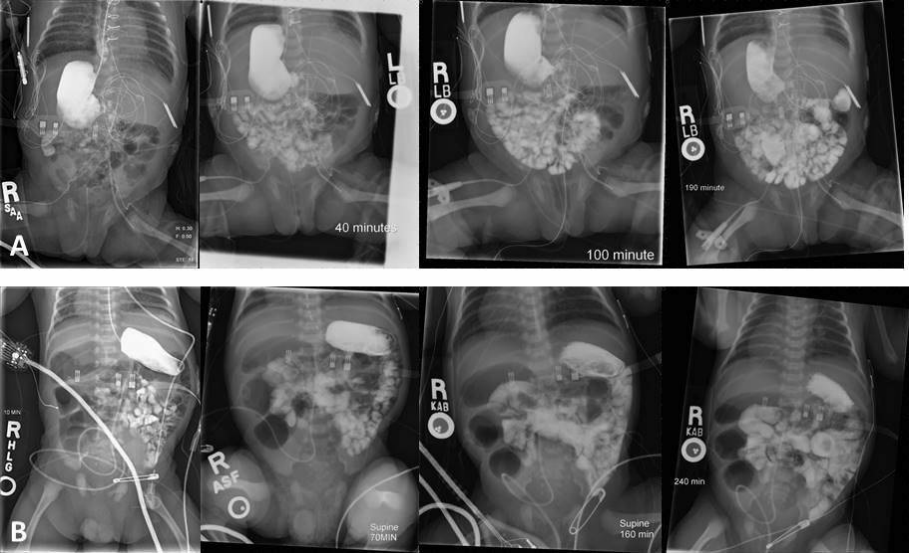
Intestinal malrotation Abdominal radiographs from upper gastrointestinal series from two separate patients with malrotation without obstruction.

Evaluation of the upper gastrointestinal tract, nonetheless, should always be undertaken when indicated (Figure 6). Interpretation, of course, requires an understanding of the varieties of malrotation. One classification suggests that rotation can be normal, mirror-imaged, exhibit a low position of the duodenojejunal junction with a wide mesentery, or exist with or without volvulus [37, 39]. In one series of 83 patients, 31% had normal rotation, with none undergoing a Ladd’s procedure, 48% had malposition of the duodenojejunal junction, with 32% undergoing an LADD's procedure, 9% had malrotation without volvulus, with 90% of  these undergoing a Ladd’s procedure, and 2% had malrotation with volvulus, all of these recommended for the Ladd’s procedure. In 14%, there was a mirror-imaged intestinal rotation, with none of these undergoing a Ladd’s procedure [40]. The duodenojejunal junction is not necessarily abnormally positioned in all the types of malrotation. There is a lower risk of volvulus, however, when the junction is positioned to the left of the midline [41]. Both the type of malrotation and the position of the duodenojejunal junction, therefore, are of importance when determining clinical decisions.

Pancreatic abnormalities are also associated with heterotaxy and include annular pancreas and partial agenesis, although their frequency is currently unknown [42-44]. Screening for pancreatic malformations does not need to be done in the absence of symptoms. Agenesis of the pancreas will more than likely be found incidentally on abdominal computed tomography. Further delineation, if needed, can be done via endoscopic retrograde cholangiopancreatography or magnetic retrograde cholangiopancreatography.

### Neurologic abnormalities

Neurologic abnormalities are known to be part of heterotaxy, although the associations are not completely understood. Agenesis of the corpus callosum, holoprosencephaly, myelomeningocele, and neural tube defects have been reported [45-46]. Such associations were also found in the mouse model of heterotaxy [47]. Discussion of these abnormalities is beyond the scope of our review. 

## Conclusions

So-called “heterotaxy” involves multiple systems. Labels, such as “asplenia” or “polysplenia”, are no longer adequate to account for the overall constellation of findings, nor is labeling in terms of “situs ambiguous”. It is better simply to describe each system in explicit detail. This allows for anticipation of functional abnormalities, as well as any interventions that may be deemed necessary. From the stance of intracardiac lesions, the distinction of right as opposed to left isomerism will permit better comparison between patients, and will point to the need specifically to address the morphology of the atrial appendages. Such a distinction is most important when assessing genotypic variations.
